# Can a physical activity program improve functional capacity and fatigue in people with cancer? A retrospective analysis

**DOI:** 10.1186/s13102-025-01066-w

**Published:** 2025-02-06

**Authors:** Aline Reinmann, Edouard Laré, Anne-Violette Bruyneel, Joseph Gligorov, Alexandre Bodmer, Thibaud Koessler

**Affiliations:** 1 Geneva School of Health Sciences, HES-SO University of Applied Sciences and Arts Western Switzerland, Rue Des Caroubiers 25, CH-1227 Carouge, Geneva, Switzerland; 2 INSERM, Centre de Recherche Saint Antoine, Sorbonne University, CRSA, 75012 Paris, France; 3 Service of Oncology, Geneva University Hospitals, Geneva, Switzerland; 4 Department of Oncology, Sorbonne University, Tenon Hospital, AP-HP Paris, France; 5 University of Geneva, Geneva, Switzerland

**Keywords:** Cancer, Physical activity, Exercise therapy, Walking, Fatigue, Education

## Abstract

**Purpose:**

The primary aim was to determine the effect of a physical activity (PA) program with education sessions on walking capacity and fatigue in people with cancer. The secondary objective was to assess the factors that moderated the program’s effect on walking capacity and fatigue among sociodemographic, physical capacity and symptom-related factors. Satisfaction with the program was also evaluated.

**Method:**

A retrospective, observational study of data from a 12-week program of twice-weekly group PA sessions combined with education sessions was conducted. The 6-min walk test (6MWT), the Multidimensional Fatigue Inventory (MFI-20) and program satisfaction were assessed. Paired t-tests were applied to assess changes in 6MWT and MFI-20. Multiple linear regressions were applied to determine the influence of age, gender, initial walking capacity and fatigue on the effects of the program.

**Results:**

Among the 264 participants (age 57.36 ± 12.43 years; 189 women; 134 with breast cancer), 125 (47%) completed the program. Walking capacity (+ 41.63 ± 91.00 m) and fatigue (-2.01 ± 3.77) were improved after the program (*p* < 0.001). Age and gender did not influence the program’s effect; however, lower initial walking capacity and higher fatigue scores were associated with larger improvements after the program. Satisfaction with the program was high among participants who completed it.

**Conclusions:**

Walking capacity and fatigue improved significantly after the PA program, but the drop-out rate was high. The program could be individualized based on an individual's initial walking capacity or fatigue score to enhance its effectiveness.

**Supplementary Information:**

The online version contains supplementary material available at 10.1186/s13102-025-01066-w.

## Introduction

Diminished physical fitness and fatigue are frequent consequences of cancer and cancer treatments [1, 2]. Physical fitness is defined as “a set of attributes that people have or achieved that relates to the ability to perform physical activity” [3]. It includes health-related fitness (e.g. cardiorespiratory endurance and muscular strength) and skill-related fitness (e.g. postural control). Both these types of fitness are affected by cancer. Maximal oxygen uptake is decreased up to 20% in people with breast cancer compared to women of the same age not affected by cancer [2]. Loss of muscle mass and function cause impairment of force production [4]. Treatment-related somatosensory deficits can cause postural control disorders [5]. These physical fitness disorders can alter physical function, which is the “physical capacity to perform a functional activity required in daily life” [6], such as walking [7, 8]. Thus, people with cancer adopt a conservative gait pattern that includes decreased gait speed, stride/step length, and power output as well as increased stride/step time, double support time, and step width variability [5, 9]. Diminished physical function associated with other negative cancer-related side effects like fatigue are problematic as they can restrict participation in PA, which in turn leads to general physical deconditioning, loss of independence in activities of daily living and reduced quality of life [9, 10].

PA is essential to counteract the repercussions of altered physical fitness. It has been extensively evaluated in phase I to III studies with different contexts (hospital, rehabilitation center, outpatient clinic; supervised or unsupervised), diverse interventions (aerobic, resistance, combined), protocols (frequency, intensity, time, type of activity [FITT]), and timings of delivery [11]. The results revealed that whatever the FITT modalities or timing of PA delivery, PA effectively improved physical function [12–14], quality of life [13] and fatigue [15]. By enhancing physical fitness components such as strength, power, balance, and maximal oxygen uptake [16–18], physical activity can improve walking capacity, as evidenced by increased distances on the 6-min walk test (6MWT) following participation in exercise programs [16]. PA can also enhance empowerment and reduce stress as well as reducing recurrence rate and increasing survival [19].

Despite the established benefits of PA, 78% of people with cancer are not sufficiently active [20]. Only 15% of people with cancer reached the PA cancer guidelines of at least 150 min of moderate to vigorous PA per week [21]. The American College of Sports Medicine guidelines recommend performing aerobic or combined training three times weekly at moderate intensity, for 12 weeks, to improve physical function and fatigue [22]. Combining therapeutic education sessions with the PA program is also suggested [23]. Despite these recommendations, people with cancer decrease their PA by up to two hours per week after the diagnosis [24]. Barriers to PA are multiple and include physiological factors (relating to the cancer and side effects of treatment), psychosocial and cultural factors (self-efficacy and motivation), and economic and environmental factors (cost, weather and access to facilities) [25].

To facilitate access to PA, many hospitals, clinics, and community-based centers offer specific supervised programs that include aerobic and resistance exercises [26, 27]. Implemented in everyday practice, these programs face the realities of clinical settings (more heterogeneous populations, practical challenges) that can influence their effectiveness. Therefore, the effectiveness of programs conducted in clinical practice should also be assessed through phase IV studies [28]. These studies aim to evaluate existing practices to identify necessary adaptations and propose effective, evidence-based, and specific interventions. To achieve this, they focus on identifying program moderators. Moderators are defined as factors present before the therapeutic intervention that can impact the treatment [29]. These factors help determine for whom and under what conditions the intervention will be effective [29]. Regarding rehabilitation programs, the effectiveness of these programs could depend on a variety of factors such as sociodemographic factors (age, gender, body mass index [BMI] and marital status), cancer-related factors (type of cancer, timing of interventions and treatment), and initial levels of fatigue and physical capacity. A lower initial PA level may result in greater benefits from the PA program (larger increase in 6MWT distance) [30] but further studies are needed to confirm this [13]. Similarly, further studies are needed to assess whether initial fatigue level influences PA response [13]. Sociodemographic and cancer-related factors seem to have only small influences on the effects of PA [13–15]. Defining moderators that promote better outcomes in PA programs will help to better target programs, better individualize future PA programs, and thus improve supportive care for people with and after cancer.

The primary aim of this retrospective study was to assess the effect of a PA program associated with educational sessions on walking capacity and fatigue in people with cancer. The secondary aim was to evaluate moderators of the program’s effect on walking capacity and fatigue among sociodemographic (age and gender), physical capacity (walking capacity) and symptom-related (fatigue) factors. We also assessed participant satisfaction at the end of the program.

## Methods

### Participants

To participate in the PA program, participants had to be at least 18 years old and have or have had an oncological disease with a medical prescription from the oncologist authorizing participation in the rehabilitation program at the Geneva University Hospital (HUG). They were excluded if they had medical conditions restricting participation in the program, such as bone metastasis or osteoporosis with a risk of fracture, verified by an oncologist. Active cancer treatments (chemotherapy, hormonal therapy, radiotherapy) were not exclusion criteria.

### Design, data source and variables

We conducted a retrospective study of data collected from September 2017 to July 2019 by physiotherapists for all the people who attended the rehabilitation program. Sociodemographic variables (age, gender, weight, height, BMI, type of cancer and treatment) were collected before the program, while clinical data (walking capacity, fatigue, and satisfaction) were collected both before and after the program.

### Rehabilitation program description

The program consisted of twice-weekly group physiotherapy sessions and three education sessions held at the HUG. Groups consisted of 10–15 participants with a diagnosis of cancer. The group program lasted 12 weeks and was supervised by two physiotherapists specialized in oncology. The education sessions consisted of presentations by healthcare professionals followed by discussions with participants. Topics covered included: PA, cancer and treatment, and nutrition. Concerning the PA program, one weekly session consisted of circuit training to work on strength, coordination, and balance, and the second weekly session involved endurance training. Circuit training sessions lasted one hour and included a 15 min warm-up and a 15 min cool down. The circuit training was composed of 15 stations of 1 min exercise and 1 min rest. The exercises, which included bodyweight, elastic band, and free weight training, varied each week to enhance motivation. Three difficulty levels were offered for each exercise. The endurance sessions involved either an easy to moderate 60-min outdoor walk or a moderate to difficult 90-min walk, or indoor training on endurance equipment (stationary bicycle ergometer, treadmill, elliptical machine), depending on each participant’s abilities. The target was to reach moderate to vigorous exercise intensity (5/10 on the BORG scale) during all sessions (0-no effort to 10-maximum effort) as recommended [22]. Each participant adapted their effort to their state of health and skills on the day. Thus, the starting level of exercise and progression were established according to the perceived exertion. Adherence rate was not monitored. Any adverse events would have been reported to the oncologist in charge of the patient.

### Evaluations

During the first and the last weeks of the program, participants took part in an evaluation session during which walking capacity and fatigue were assessed by the physiotherapists who also performed the rehabilitation.

#### The 6-min walk test (6MWT)

6MWT is a very common test of exercise capacity used in oncology [31]. It is moderately to strongly correlated with other walking capacity tests such as the 2-min walking test and the 10-m walk test in individuals with cancer (0.61 > *r* > 0.84, *p* < 0,001) [32] and has excellent test–retest reliability (ICC = 0.96) [33].

The 6MWT was conducted according to the guidelines of the European Respiratory Society / American Thoracic Society [34]. The participants had to walk back and forth along a 30 m corridor aiming to walk ‘as far as possible in six minutes’. No incentives were given. After six minutes, participants were asked to stop, and the distance walked was measured by means of markers on the wall. Heart rate (HR), oxygen saturation (SpO^2^) measured by a fingertip pulse oximeter, perceived exertion (BORG) and the walking distance were collected before, every two minutes, and at the end of the test. The time to recover HR and SpO^2^ (return to baseline values) was also recorded.

#### The Multidimensional Fatigue Inventory (MFI-20) questionnaire

The MFI-20 is a 20-item questionnaire that assesses the influence of cancer on general physical and mental fatigue, activity, and motivation [35]. Subscale scores range from 4 to 20 with higher scores indicating higher fatigue. This questionnaire is validated in French (*r* = 0.83) and is highly reliable (Cronbach’s alpha coefficient = 0.80) [35, 36]. It is recommended in an oncological context where assessment of fatigue is an important aspect of patient monitoring [36].

#### Satisfaction questionnaire

The satisfaction questionnaire was designed by the HUG to assess participant satisfaction with the rehabilitation program (Supplementary file). It includes questions about hospital choice, the program admission process, the program, and facilities that are rated on a Likert scale from 1 (excellent) to 5 (poor) and 6 (don't know) (range 14–82) (Table 3). This questionnaire was completed at the end of the program.

### Statistical analysis

Descriptive statistics were expressed as frequencies and percentages for qualitative variables (sex, type of cancer, treatment, and satisfaction) and mean ± standard deviation for continuous variables (age, height, body weight, BMI, functional capacities, and fatigue results).

For the primary objective, paired t-tests were used to assess change in 6MWT distance and change in fatigue from the start to the end of the PA program after confirming the normality of the distribution of the differences with an exploratory data analysis. Cohen’s d effect sizes were calculated.

To assess whether sociodemographic factors, baseline level of PA, and baseline fatigue moderated the effects of the PA program, multiple linear regression analyses were conducted. In these models, the evolution of the dependent variable (DV, either walking capacity or fatigue) was considered as a function of the initial parameter values (IV), with age and gender included as covariates. The assumptions of the model, including linearity of the DV *vs.* IV, normality and homoscedasticity of residuals, were verified. Multiple linear regression analyses were preferred over mixed models because the mixed models had non-significant likelihood ratio tests, minimal variances of the random effects, and higher Akaike Information Criterion (AIC) and Bayesian Information Criterion (BIC) values compared to the multiple linear regressions.

Analyses were conducted with Stata software (v.15, Stata Corporation, USA; v.25) and a *p*-value < 0.05 was considered significant for all analyses.

## Results

### Socio-demographic characteristics

A total of 264 participants, mean age 57.36 $\pm$ 12.43 years underwent the baseline assessment (Table 1). Breast cancer was by far the most represented cancer (51%, *n* = 134) followed by lung (9%, *n* = 23) and colorectal cancers (7%, *n* = 18).

**Table 1:** Participant characteristics

	**All participants**		**Participants who completed the program**		**Participants who dropped out of the program**		
**Characteristics**	Values	*n*	Values	*n*	Values	*n*	*p*-value finishers *vs.* drop-out
**Age, (years)**	57.36 ± 12.43 (18–89)	263	58.21 ± 11.49 (31–89)	125	56.59 ± 13.22 (18–88)	138	0.292
Age women	56.22 ± 12.17 (18–88)	188	56.63 ± 11.13 (31–81)	99	55.76 ± 13.28 (18–88)	89	0.629
Age men	60.21 ± 12.69 (23–89)	75	64.23 ± 11.00 (40–89)	26	58.08 ± 13.11 (23–74)	49	**0.045**
**Gender, n (%)**		264		125		139	
Women	189 (72)	189	99 (79)	99	90 (65)	90	
Men	75 (28)	75	26 (21)	26	49 (35)	49	
**Height, (cm)**	164.93 ± 9.61 (147–202)	149	163.17 ± 8.17 (147–186)	69	166.45 ± 10.51 (150–202)	80	**0.037**
Height women	160.76 ± 6.56 (147–178)	106	160.67 ± 6.43 (147–176)	55	160.84 ± 6.76 (150–178)	51	0.895
Height men	175.23 ± 8.05 (163–202)	43	173.00 ± 6.82 (163–186)	14	176.31 ± 8.48 (163–202)	29	0.210
**Weight, (kg)**	73.63 ± 18.09 (42–174)	150	71.57 ± 16.42 (42–120)	69	75.38 ± 19.33 (43–174)	81	0.199
Weight women	69.29 ± 16.24 (42–105)	107	67.80 ± 14.96 (42–102)	55	70.88 ± 17.50 (43–105)	52	0.330
Weight men	84.41 ± 18.14 (60–174)	43	86.36 ± 13.62 (67–120)	14	83.47 ± 20.12 (60–174)	29	0.630
**BMI, (kg/m2)**	27.04 ± 5.97 (16.16–58.14)	149	26.74 ± 5.13 (16.16–39.35)	69	27.30 ± 6.64 (17.16–58.14)	80	0.569
BMI women	26.85 ± 6.02 (16.16–45.33)	106	26.21 ± 5.31 (16.16–39.35)	55	27.54 ± 6.70 (17.16–45.33)	51	0.259
BMI men	27.52 ± 5.88 (19.82–58.14)	43	28.83 ± 3.82 (20.68–34.69)	14	26.89 ± 6.63 (19.82–58.14)	29	0.317
**Cancer type, n (%)**		264		125		139	
Appendix	1 (< 1)		1 (1)		0		
Bladder	2 (1)		1 (1)		1 (1)		
Bone	1 (< 1)		0		1 (1)		
Breast	134 (51)		76 (61)		58 (42)		
Brain	9 (3)		3 (2)		6 (4)		
Brain lymphoma	1 (< 1)		0		1 (1)		
Colorectal	18 (7)		9 (7)		9 (7)		
Gall bladder	2 (1)		1 (1)		1 (1)		
Gynecological	14 (5)		4 (3)		10 (7)		
Kidney	3 (1)		1 (1)		2 (1)		
Leukemia	4 (2)		3 (2)		1 (1)		
Liver	3 (1)		1 (1)		2 (1)		
Lung	23 (9)		8 (6)		15 (11)		
Lymphoma	8 (3)		2 (2)		6 (4)		
Mediastinum	1 (< 1)		0		1 (1)		
Oesophagus	2 (1)		0		2 (1)		
ORL	6 (2)		2 (2)		4 (3)		
Pancreas	6 (2)		2 (2)		4 (3)		
Prostate	11 (4)		6 (6)		5 (4)		
Skin	4 (2)		2 (2)		2 (2)		
Small intestine	1 (< 1)		0		1 (1)		
Spleen	1 (< 1)		1 (1)		0		
Stomach	7 (3)		2 (2)		5 (4)		
Thyroid	2 (1)		0		2 (1)		
**Therapy, n (%)**		232		105		127	
C	39 (15)		15 (14)		24 (19)		
C + S	26 (10)		10 (10)		16 (13)		
C + S + H	2 (1)		1 (1)		1 (1)		
C + S + H + R	9 (3)		6 (6)		3 (2)		
C + S + I	2 (1)		1 (1)		1 (1)		
C + S + I + R	5 (2)		2 (2)		3 (2)		
C + S + R	34 (13)		19 (18)		15 (12)		
C + H	4 (2)		2 (2)		2 (2)		
C + H + I	1 (< 1)		0		1 (1)		
C + H + R	10 (4)		5 (5)		5 (4)		
C + I	4 (2)		0		4 (3)		
C + I + R	2 (1)		1 (1)		1 (1)		
C + R	26 (10)		8 (8)		18 (14)		
S	13 (5)		9 (9)		4 (3)		
S + H	6 (2)		4 (4)		2 (2)		
S + H + I + R	1 (< 1)		1 (1)		0		
S + H + R	14 (5)		9 (9)		5 (4)		
S + I	1 (< 1)		0		1 (1)		
S + R	12 (5)		4 (4)		8 (6)		
H	4 (2)		2 (2)		2 (2)		
H + R	5 (2)		1 (1)		4 (3)		
I	8 (3)		3 (3)		5 (4)		
R	1 (< 1)		0		1 (1)		
None	3 (1)		2 (2)		1 (1)		

Socio-demographics and clinical characteristics of participants. Data are mean ± SD (Min–Max) or n (%)

*BMI* Body Mass Index, *C* Chemotherapy, *H* Hormone therapy, *I* Immune therapy, *ORL* Otorhinolaryngological, *R* Radiation therapy, *S* Surgery

In bold, *p*-value < 0.05

### Study completion

Among the 264 participants, 47% finished the program. Data of 139 persons were missing (drop-out or methodological reasons). The reasons for drop out were not monitored. No major adverse events occurred during the program.

The initial walking distance of individuals who did not complete the program was not different from the initial walking distance of those who finished it (mean ± SD (min–max): 475.88 ± 120.82 (120 – 720) *vs.* 487.98 ± 100.30 (120—780), *p* = 0.389). The same result was found for general fatigue (13.33 ± 3.74 (4 – 20) *vs.* 12.88 ± 3.74 (4 – 20), *p* = 0.335).

### Changes in 6MWT distance

6MWT distance improved by 41.63 ± 91.00 m (+ 9%, *p* < 0.001, *d *= 0.41, *n* = 121) from the start to the end of the program (Table 2). BORG, HR, SpO^2^ values, and recovery time did not change.

**Table 2:** Descriptive characteristics, functional, and fatigue values

	Values pre	*n*	Values post	*n*	Differences	*n*	Cohen’s *d* effect size	*p* -value
*Walking capacity with the 6MWT*								
Distance 2 min, (m)	163.40 ± 29.10 (90—250)	119	176.72 ± 33.89 (90—290)	118	13.46 ± 26.61 (−75—90)	118	0.43	** < 0.001**
Distance 4 min, (m)	323.57 ± 64.15 (100—500)	119	349.25 ± 67.99 (150—540)	118	25.98 ± 60.74 (−180—210)	118	0.39	** < 0.001**
Distance 6 min, (m)	487.98 ± 100.30 (120—780)	121	529.60 ± 102.56 (180—835)	121	41.63 ± 91.00 (−330—375)	121	0.41	** < 0.001**
BORG 6 min	3.87 ± 1.92 (0.5—9)	108	3.95 ± 1.87 (1—10)	57	−0.12 ± 2.18 (−5—6)	52	−0.06	= 0.704
SpO^2^ pre, (%)	97.45 ± 1.35 (93—100)	121	97.18 ± 1.51 (93—100)	120	−0.25 ± 1.62 (−6—3)	120	−0.18	= 0.094
SpO^2^ 2 min, (%)	96.02 ± 1.79 (90—99)	121	95.59 ± 2.59 (85—100)	121	−0.41 ± 2.69 (−13—6)	121	−0.18	= 0.099
SpO^2^ 4 min, (%)	95.93 ± 2.15 (86—100)	121	95.50 ± 2.86 (81—100)	121	−0.43 ± 3.19 (−17—12)	121	−0.17	= 0.141
SpO^2^ 6 min, (%)	96.18 ± 1.88 (90—100)	121	95.74 ± 2.47 (87—100)	121	−0.44 ± 2.76 (−12—8)	121	−0.20	= 0.083
HR pre, (bpm)	81.64 ± 13.60 (51—123)	121	86.60 ± 13.49 (57—120)	121	4.96 ± 12.71 (−21—56)	121	0.37	** < 0.001**
HR 2 min, (bpm)	112.02 ± 19.42 (62—171)	120	111.65 ± 19.80 (48—153)	120	−0.49 ± 23.56 (−78—61)	119	−0.03	= 0.822
HR 4 min, (bpm)	114.05 ± 19.25 (60—173)	119	115.43 ± 19.59 (62—156)	120	0.92 ± 19.26 (−77—59)	118	0.05	= 0.603
HR 6 min, (bpm)	116.65 ± 19.44 (63—165)	121	118.02 ± 20.27 (63—165)	121	1.37 ± 17.60 (−86—58)	121	0.07	= 0.393
Recuperation SpO^2^ 6 min, (s)	48.27 ± 57.40 (0—265)	115	43.23 ± 49.10 (0—180)	115	−3.62 ± 73.62 (−185—150)	109	−0.18	= 0.608
Recuperation HR 6 min, (s)	123.54 ± 59.83 (0—360)	115	109.19 ± 54.22 (0—180)	115	−9.91 ± 74.05 (−360—180)	109	−0.07	= 0.165
*Fatigue with the MFI-20*								
General fatigue	12.88 ± 3.74 (4—20)	121	10.87 ± 3.74 (4- 20)	121	−2.01 ± 3.77 (−11—6)	121	−0.55	** < 0.001**
Physical fatigue	12.45 ± 4.32 (4—20)	121	9.91 ± 4.07 (4—20)	121	−2.54 ± 4.05 (−14—11)	121	−0.58	** < 0.001**
Reduced PA	11.51 ± 4.33 (4—20)	121	9.48 ± 3.81 (4—20)	121	−2.03 ± 3.89 (−13—8)	121	−0.48	** < 0.001**
Reduced motivation	8.58 ± 3.26 (4—18)	121	7.79 ± 3.05 (4—16)	121	−0.79 ± 3.10 (−8—7)	121	−0.24	** = 0.008**
Mental fatigue	10.11 ± 4.29 (4—20)	121	9.03 ± 4.24 (3—20)	121	−1.07 ± 3.63 (−9—11)	121	−0.24	** = 0.003**

Descriptive characteristics, functional, and fatigue values

Data are mean ± SD (Min–Max)

*HR* Heart Rate, *SpO* ^*2*^ Oxygen saturation, *6MWT* 6-min walk test

In bold, *p*-value < 0.05

### Changes in fatigue

All the dimensions of the MFI20 questionnaire were improved after the program (Table 2). General and physical fatigue, reduced PA, reduced motivation, and mental fatigue were decreased by −2.01 ± 3.77 (−16%, *p* < 0.001, *d* = −0.55, *n* = 121), −2.54 ± 4.05 (−20%, *p* < 0.001, *d* = −0.58, *n* = 121), −2.03 ± 3.89 (−18%, *p* < 0.001, *d* = 0.48, *n* = 121), −0.79 ± 3.10 (−9%, *p* = 0.008, *d* = −0.24, *n* = 121), and −1.07 ± 3.63 (−11%, *p* = 0.003, *d* = −0.24, *n* = 121).

### Changes in the 6MWT according to age, gender, and initial walking distance

The change in walking distance was not influenced by age (*p* = 0.415) or gender (*p* = 0.093) but did vary based on the initial walking distance value (*p* < 0.001, Table 3). Multiple linear regression analysis indicated that for each additional meter of initial walking distance, the change in walking distance after the program would decrease by 0.43 m. The *p-value* of the multiple linear regression was significant (*p* < 0.001).

**Table 3:** Linear regressions

	Simple linear regression coefficient	95% CI	*p*—value	Multiple linear regression coefficient	95% CI	*p*—value
*Walking model*						
Initial walking distance	−0.389	−0.54 to −0.24	** < 0.001**	−0.429	−0.59 to −0.27	** < 0.001**
Age	0.900	−0.55 to 2.35	0.222	−0.601	−2.06 to 0.86	0.415
Gender	14.739	−27.10 to 56.58	0.487	34.272	−5.78 to 74.33	0.093
*Fatigue model*						
Initial general fatigue	−0.509	−0.67 to −0.35	** < 0.001**	−0.505	−0.67 to −0.34	** < 0.001**
Age	0.039	−0.02 to 0.10	0.198	0.014	−0.04 to 0.07	0.627
Gender	0.464	−1.22 to 2.15	0.586	−0.260	−1.80 to 1.28	0.739

Coefficients of linear regressions *n* = 121

*CI* Confidence interval

In bold, *p*-value < 0.05

### Changes in general fatigue according to age, gender, and initial fatigue

Change in general fatigue did not differ according to age (*p* = 0.627) or gender (*p* = 0.739) but it did differ according to the initial general fatigue value (*p* < 0.001, Table 3). The change in fatigue score after the program would decrease by 0.51 points for each additional point in the initial fatigue score.

### Satisfaction

Participants were particularly satisfied, both in terms of the location of the program and the program itself (Table 4): 91% stated they would recommend it “without a doubt” and 9% “probably”.

**Table 4:** Satisfaction questionnaire

*Choice*						
	*Me*	*My doctor*	*Advice from a relative*			
Hospital choice for your rehabilitation (*n* = 111)	18 (16.22)	90 (81.08)	3 (2.7)			
*Facilities*						
	*Excellent*	*Very good*	*Good*	*Fair*	*Poor*	*I don’t know*
Easy access to the Physiotherapy Department (*n* = 116)	69 (59.48)	38 (32.76)	7 (6.03)	1 (0.86)	1 (0.86)	0
Ease of orientation around and in buildings (*n* = 114)	63 (55.26)	38 (33.33)	13 (11.4)	0	0	0
Comfort, cleanliness, lighting, temperature of room in which you were treated (*n* = 116)	69 (59.48)	30 (25.86)	14 (12.07)	3 (2.59)	0	0
Calm, intimacy, relaxing atmosphere of the rehabilitation rooms (*n* = 113)	59 (52.21)	38 (33.63)	16 (14.16)	0	0	0
*Admission*						
Ease of formalities and time to get your first appointment (*n* = 114)	76 (66.67)	33 (28.95)	5 (4.39)	0	0	0
Friendliness, willingness of staff to answer your questions, expectations (*n* = 116)	69 (59.48)	37 (31.9)	8 (6.9)	2 (1.72)	0	0
*Your treatment*						
Ability of your physiotherapist to make you feel comfortable and, if necessary, reassure you (*n* = 115)	79 (68.7)	33 (28.7)	3 (2.61)	0	0	0
Explanations you received about what would be done to you and what was expected of you (*n* = 116)	70 (60.34)	34 (29.31)	10 (8.62)	0	0	2 (1.72)
At the end of your physiotherapy, the quality of information you received about your future (*n* = 116)	49 (42.24)	37 (31.9)	18 (15.52)	1 (0.86)	0	11 (9.48)
Your sense of security at each stage of rehabilitation (*n* = 117)	78 (66.67)	33 (28.21)	6 (5.13)	0	0	0
Tailoring your rehabilitation to the specificity of your problem (*n* = 113)	55 (48.67)	40 (35.4)	18 (15.93)	0	0	0
*Summary word*						
If you had to summarize your physiotherapy treatment in one word (*n* = 111)	65 (58.56)	40 (36.04)	6 (5.41)			
*Recommendation*						
	*Yes, without any doubt*	*Yes, probably*				
Would you recommend our service to someone close to you? (*n* = 116)	105 (90.52)	11 (9.48)				

Results of the satisfaction questionnaire. Data expressed in n (%). *n* = 111—117

## Discussion

This retrospective study showed that at the end of a PA exercise program combined with education sessions, walking distance and all dimensions of fatigue in people with different types of cancer had improved (small to moderate effect sizes [37]). However, the drop-out rate was high. Low walking capacity and high fatigue levels were associated with greater benefits from the program, while age and gender had no influence on the results. Participants who completed the program were highly satisfied with it.

The improvement in walking capacity following the PA program is consistent with previous work. Systematic reviews found that PA can improve aspects of physical fitness, such as strength, power, balance and maximal oxygen uptake, in people with different types of cancer [16–18]*,* as well as walking capacity, with improvements in the 6MWT, 400 m walk, 6 m or 10 m walk tests [17, 18, 38]*.* However, the improvement in the 6MWT distance did not always exceed the minimum clinically significant difference (MCID) [30, 39] established at 42.7 m [40]*,* 42 m or 9,5% improvement [41], 63.2 m [42], or 62.5 m [39] in studies of people with prostate, lung, and breast cancer. In the study by Soucy et al. (2022), the MCID was reached in only 28% of participants [30]. Similarly, in our study, the improvement in 6MWT distance was close to, but did not exceed the MCID, suggesting that providing more individualized PA programs, particularly regarding the participant's initial condition, could improve its effectiveness.

The participants with a shorter baseline 6MWT distance had greater improvements in walking capacity than those with a longer baseline 6MWT distance. It is possible that these results are related to the greater sensitivity of the test to functional changes in cases of lower initial capacities [43]. Another explanation concerns the margin for progress. Individuals starting from a lower physical capacity likely have a greater potential for improvement [44]. Thus, in a previous study evaluating the effects of a community program in women with breast cancer, an improvement in walking capacity, assessed using the 6MWT, was observed in individuals with lower initial capacities [30]. This confirms the necessity of providing individualized PA programs designed according to the individual’s physical capacities, such as walking capacity, rather than according to sociodemographic factors, since these factors did not influence the improvement in walking distance in this study. A similar lack of influence was also found in a meta-analysis [13].

In addition to considering initial condition, an objective assessment of maximal capacities could enhance program effectiveness by accurately prescribing the appropriate starting load and progression. Individuals with high initial capacities, whose range of gains is consequently more limited, could benefit from more individualized stimuli, either in a more objective manner [44] or more frequently, as recommended [22]. This would address the principles of progression, overload, and specificity, that must be adhered to for the effective implementation of rehabilitation programs [44].

The addition of education sessions by trained caregivers, as was done in this program, is another method to increase the effectiveness of PA programs by supporting and guiding cancer patients in their engagement with physical activity [25, 45].

The results for fatigue were similar to most results of the latest systematic reviews and meta-analyses that found that PA improved cancer-related fatigue in people with breast [10, 46], gynecological [47], lung [48], colon [49–51], and pancreatic [18] cancer. PA induces anti-inflammatory effects on IL-6 that may reduce the activity of pro-inflammatory cytokines IL-1B and TNF-a, positively affecting cancer-related fatigue [52]. All the dimensions evaluated by the fatigue questionnaire were improved, namely general fatigue, physical fatigue, PA, motivation, and mental fatigue, but only the change in physical fatigue exceeded the MCID (2.54 > 2.04), although the change in general fatigue was very close to the MCID (2.01 < 2.06) [53]. A meta-analysis found that during cytotoxic treatment, general fatigue and physical fatigue were also improved by PA [54]. Therefore, PA may specifically improve these fatigue dimensions.

PA seemed particularly beneficial for people with high initial level of fatigue. Participants with higher level of fatigue had greater improvements in their MFI20 scores at the end of the program than those with lower levels of baseline fatigue. On the opposite, age and gender had no influence on the program's effect on fatigue in this retrospective study, contrary to what was observed in Covington's scoping review (2019) [26]. Knowing that a PA program with education sessions can reduce cancer-related fatigue, particularly high levels of fatigue could encourage people with cancer to participate in PA [25]. Indeed, treatment-related side effects, medical complications, and cancer progression are among the main barriers to PA, along with time conflicts [26]. The fact that some participants were receiving active oncological therapy such as chemotherapy at the same time as the PA program could partly explain the high dropout-rate found in this study. As many of the adverse effects of disease and treatment can be ameliorated by PA [55], there is a need to find ways to facilitate adherence to PA to help people to achieve the recommendations. Quality therapeutic education provided by trained caregivers, the implementation of PA as an integral part of cancer treatment, the reorganization of health-care facilities or the diversification of the modalities of the individualized program offered (at home, with different types of PA, etc.), could be possible solutions [25].

### Limitations

This study was based on retrospective data, with the disadvantages that this entails. Thus, some demographic and outcome data were missing. Additional information on the participants, e.g. cancer stage, PA practiced outside the program, adherence in terms of quantity and intensity of exercise performed during PA, and other physical fitness capacities affected by cancer, such as strength or postural control, would have provided additional information on the effects of a PA program. Similarly, the addition of a control group would have allowed confirmation that the observed improvements were due to the program. Evaluations at several time points would have increased the accuracy of the results by knowing how they evolve throughout the program [30, 44]. Knowledge on missing data and reasons of drop-out would have been of great interest to set up future physical activity programs. As the physiotherapists in charge of the program administered the satisfaction questionnaire, this may have led to a social desirability bias and thus overestimating program satisfaction. Finally, the small number of participants who completed the program prevented further analyses, for example by type of cancer. However, previous studies did not demonstrate any influence of cancer type on PA program effects on physical function [13] or fatigue [15].

### Practical implications and conclusions

This retrospective study found improvements in walking capacity and fatigue among people with cancer who completed the hospital-based PA program. To enhance its effectiveness, the program could be individualized based on the individual's initial condition, specifically their initial walking capacity and fatigue levels. Lower walking capacity and higher fatigue levels resulted in greater benefits. Particular attention should be paid to reducing missing data and drop-out rates when developing a PA program. The high level of satisfaction reported by participants who completed the program demonstrated the potential to offer appropriate exercise programs during the active phase of treatment.

## Supplementary Information

Supplementary Material 1.

## Data Availability

The data that support the findings of this study will be made available by the authors upon reasonable request.

## Abbreviations

6MWT: 6-min walk test
BMI: Body Mass Index
FITT: Frequency, Intensity, Time, Type of activity
HR: Heart Rate
HUG: Geneva University Hospital
IL-1B: Interleukine-1 beta
MCID: Minimal Clinically Important Difference
MFI-20: Multidimensional Fatigue Inventory
PA: Physical Activity
SpO^2^: Oxygen saturation
TNF-a: Tumor Necrosis Factor-alpha
