# Supplementary material for: Can a physical activity program improve functional capacity and fatigue in people with cancer? A retrospective analysis
Source: BMC Sports Sci Med Rehabil. 2025 Feb 6;17:21. doi: 10.1186/s13102-025-01066-w (PMC11800585; doi:10.1186/s13102-025-01066-w)
Supplement: Supplementary file 1 — Supplementary Material 1. [file 13102_2025_1066_MOESM1_ESM.docx]

**Journal: BMC Sports Science, Medicine and Rehabilitation**

**Can a physical activity program improve functional capacity and fatigue in people with cancer? A retrospective analysis**

**Supplementary file**

1. *Satisfaction questionnaire*

| *Choice* | *Me* | | *My doctor* | | *Advice from a relative* | | | *No other choice* | |
| --- | --- | --- | --- | --- | --- | --- | --- | --- | --- |
| Hospital choice for your rehabilitation | 1 | | 2 | | 3 | | | 4 | |
|  | | | | | | | | | |
| *Facilities* | *Excellent* | *Very good* | | *Good* | | *Fair* | *Poor* | | *I don’t know* |
| Easy access to the Physiotherapy Department | 1 | 2 | | 3 | | 4 | 5 | | 6 |
| Ease of orientation around and in buildings | 1 | 2 | | 3 | | 4 | 5 | | 6 |
| Comfort, cleanliness, lighting, temperature of the room in which you were treated | 1 | 2 | | 3 | | 4 | 5 | | 6 |
| Calm, intimacy, relaxing atmosphere of the rehabilitation rooms | 1 | 2 | | 3 | | 4 | 5 | | 6 |
|  | | | | | | | | | |
|  | | | | | | | | | |
| *Admission procedure* | | | | | | | | | |
| Ease of formalities and time to get your first appointment | 1 | 2 | | 3 | | 4 | 5 | | 6 |
| Friendliness, willingness of staff to answer your questions and meet your expectations | 1 | 2 | | 3 | | 4 | 5 | | 6 |
|  | | | | | | | | | |
|  | | | | | | | | | |
| *Your treatment* | | | | | | | | | |
| Ability of your physiotherapist to make you feel comfortable and, if necessary, reassure you | 1 | 2 | | 3 | | 4 | 5 | | 6 |
| Explanations you received about what would be done to you and what was expected of you | 1 | 2 | | 3 | | 4 | 5 | | 6 |
| At the end of your physiotherapy, the quality of information you received about your future | 1 | 2 | | 3 | | 4 | 5 | | 6 |
| Your sense of security at each stage of rehabilitation | 1 | 2 | | 3 | | 4 | 5 | | 6 |
| Tailoring your rehabilitation to the specificity of your problem | 1 | 2 | | 3 | | 4 | 5 | | 6 |
|  | | | | | | | | | |
|  | | | | | | | | | |
| *Summary word* | | | | | | | | | |
| If you had to summarize your physiotherapy treatment in one word | 1 | 2 | | 3 | | 4 | 5 | | 6 |
|  | | | | | | | | | |
| *Recommendation* | *Yes, without any doubt* | *Yes, probably* | | *Maybe* | | *No, probably not* | *No, absolutely not* | | *I don’t know* |
| Would you recommend our Service to someone close to you? | 1 | 2 | | 3 | | 4 | 5 | | 6 |
| ***Supplementary file 1****. Satisfaction questionnaire* | | | | | | | | | |
